# Analysis of digital vernier caliper *versus* digital thickness gauge consistency and user experience in measuring arterial wall thickness: implications for biomechanical assessment

**DOI:** 10.3389/fbioe.2026.1791599

**Published:** 2026-05-12

**Authors:** Constantin Claudiu Ciucanu, Eliza Russu, Alexandru Mureşan, Alexandra Ioana Asztalos, Alexandru Petru Ion, Réka Bartus, Eliza-Mihaela Arbănaşi, Adrian Vasile Mureşan, Carmen Corina Radu, Timur Hogea, Traian V. Chirilă, Septimiu Toader Voidǎzan, Emil-Marian Arbănaşi

**Affiliations:** 1 Department of Vascular Surgery, George Emil Palade University of Medicine, Pharmacy, Science and Technology of Targu Mures, Targu Mures, Romania; 2 Doctoral School of Medicine and Pharmacy, George Emil Palade University of Medicine, Pharmacy, Science and Technology of Targu Mures, Targu Mures, Romania; 3 Clinic of Vascular Surgery, Mures County Emergency Hospital, Targu Mures, Romania; 4 Regenerative Medicine Laboratory, Centre for Advanced Medical and Pharmaceutical Research (CCAMF), George Emil Palade University of Medicine, Pharmacy, Science and Technology of Targu Mures, Targu Mures, Romania; 5 Department of Forensic Medicine, George Emil Palade University of Medicine, Pharmacy, Science and Technology of Targu Mures, Targu Mures, Romania; 6 Institute of Forensic Medicine, Targu Mures, Romania; 7 Faculty of Medicine, George E. Palade University of Medicine, Pharmacy, Sciences and Technology, Târgu Mureş, Romania; 8 Queensland Eye Institute, Woolloongabba, Australia; 9 Australian Institute of Bioengineering & Nanotechnology (AIBN), University of Queensland, Brisbane, Australia; 10 Department of Epidemiology, George Emil Palade University of Medicine, Pharmacy, Science and Technology of Targu Mures, Targu Mures, Romania

**Keywords:** biomechanics, digital thickness gauge, digital vernier caliper, thickness, vascular tissue

## Abstract

**Introduction:**

Accurately measuring tissue thickness is a crucial technical aspect and a source of bias in biomechanical analysis, as even minor errors can substantially affect stress, elasticity, and tissue behavior. This study analyzes the reproducibility and consistency of specimen thickness measurements by experienced users using a digital caliper under two protocols and a specialized device under a third, also assessing the impact on biomechanical properties.

**Method:**

The current study is a methodological study in which we examined the thickness of the porcine arterial wall, specifically segments from the aorta, carotid, and coronary arteries. For the first two protocols, thickness measurements were performed using a digital vernier caliper (Multicomp PRO MP012475), whereas a dedicated digital thickness gauge (Mitutoyo 547-500S, Kawasaki, Japan) was employed for the third protocol. Biomechanical testing was conducted using a BioTester® 5000 (CellScale, Canada), fitted with two opposing BioRakes mounted on actuators to apply uniaxial tensile loading at 25% and 50% stretch.

**Results:**

For coronary artery wall thickness measurements, significant protocol-dependent differences between novice users for Protocol 1 and Protocol 3 (p = 0.0384), and for intermediate users using the same protocols (p = 0.0122). The mechanical response of porcine vascular tissues at 25% and 50% stretch was not influenced by operator experience, as no statistically significant differences in Cauchy stress were observed among users across the three experimental protocols.

**Conclusion:**

This study demonstrates that the method used to measure arterial wall thickness influences reproducibility, particularly in thin-walled vessels such as coronary arteries, and when performed by less experienced users.

## 1 Introduction

Technological progress has advanced significantly in recent years, particularly in the biomedical field, where challenges remain constant. Although multiple methods are currently available to address cardiovascular pathologies, they remain a significant public health problem in both developed and developing countries (Santos et al., 2025; Teo and Rafiq, 2021; Li et al., 2023). In addition to the drug and surgical therapies currently available, it is essential to understand the mechanisms underlying the occurrence and development of cardiovascular diseases as comprehensively as possible. In this regard, there remain uncertainties in characterizing the biomechanical behavior of soft tissues, such as vascular tissue, particularly given their anisotropy (Holzapfel et al., 2004; Wang et al., 2019). To better evaluate these properties, various calculation formulas are used, including Cauchy stress and Young’s modulus. The Cauchy stress tensor describes the state of stress at a point in a deformable body and represents the forces per unit area acting on the tissue. Young’s modulus, also known as the modulus of elasticity, is a property of a material that quantifies its stiffness, specifically the ratio of stress to strain in the elastic region (Hooglugt et al., 2022; Lisický et al., 2021). These formulas are based on the cross-sectional area of the tissue being analyzed and therefore require the most appropriate collection of sample dimensions. Various tools can be used to perform these measurements, each with its own perks and drawbacks. These include digital vernier calipers, micrometers, or thickness gauges (O’Lear et al., 2013; de Gelidi et al., 2017). Recently published papers from our team show that the method used to measure tissue thickness has a substantial impact on data quality: a digital thickness gauge yields more consistent and reliable measurements than a digital vernier caliper (Ion et al., 2024a; Ion et al., 2024b). Accurately measuring tissue thickness is a crucial technical aspect and a significant source of bias in biomechanical analysis, as even minor errors can substantially affect stress, elasticity, and tissue behavior calculations.

This study aims to analyze the reproducibility and consistency of arterial wall thickness measurements obtained with a standard digital vernier caliper and a specialized digital thickness gauge across examiners with different levels of experience, and to determine whether these thickness measurements influence the derived biomechanical profile of porcine aortic, carotid, and coronary artery walls.

## 2 Materials and methods

### 2.1 Vascular tissue samples

The current study is a methodological study in which we examined the thickness of the porcine arterial wall, specifically segments from the aorta, carotid arteries, and coronary arteries. All tissues were obtained from a local slaughterhouse, as these organs were designated for disposal. To ensure optimal preservation, the harvested tissues were promptly transported to the laboratory and stored at 4 °C. Following appropriate tissue preparation, 12 segments were excised from each artery, producing samples measuring 12 × 12 mm. Each specimen was subsequently stored in phosphate-buffered saline (PBS) until further analysis.

### 2.2 Thickness measurements protocols

**FIGURE 1:**
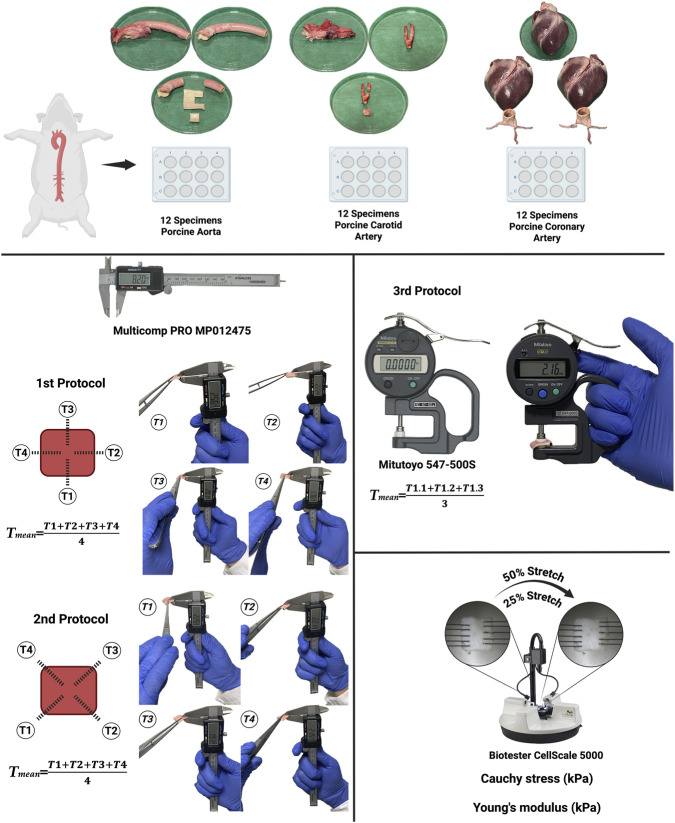
Experimental workflow for arterial wall thickness measurement and mechanical characterization. Porcine arterial specimens (n = 12 per vessel type: aorta, carotid, and coronary) were harvested and prepared for thickness assessment and biomechanical testing. Wall thickness was measured using three protocols: Protocol I (side-midpoint measurements) and Protocol II (corner measurements) using a digital vernier caliper (Multicomp PRO MP012475), and Protocol III using a digital thickness gauge with controlled contact force (Mitutoyo 547-500S). In Protocols I and II, thickness was calculated as the average of four measurements (T_1_–T_4_), whereas in Protocol III, the mean of three repeated measurements at the same location was used. Following thickness determination, specimens were subjected to uniaxial tensile testing using a CellScale BioTester 5000. Mechanical properties were quantified by calculating Cauchy stress (kPa) at 25% and 50% stretch and Young’s modulus (kPa) from the linear region of the stress–strain response.

Thickness measurements were obtained using two instruments with fundamentally different operating principles. The digital vernier caliper determines specimen thickness as the distance between two manually positioned jaws. Consequently, the recorded value is influenced by operator-dependent factors, including jaw alignment, selection of the contact location, and the magnitude of the applied force. In contrast, the digital thickness gauge employs a probe–anvil configuration with spring-regulated contact. This design delivers a consistent measuring force, thereby minimizing variability associated with tissue compression, particularly in compliant specimens, and reducing operator-dependent measurement bias. For the first two protocols, thickness measurements were performed using a digital vernier caliper (Multicomp PRO MP012475), whereas a dedicated digital thickness gauge (Mitutoyo 547-500S, Mitutoyo Corp., Kawasaki, Japan) was employed for the third protocol. Protocols I and II were designed to replicate common operator-dependent measurement strategies encountered when using a digital vernier caliper on small, square vascular specimens. Protocol I (side-midpoint measurements) models a user who preferentially positions the caliper centrally along each edge. Thickness was measured at the midpoint of each side, and the resulting values were averaged to obtain a representative specimen thickness. Protocol II (corner measurements) reflects an alternative approach in which the caliper is positioned near specimen corners or transition zones. These regions are more susceptible to local geometric variability, including edge curvature, minor surface irregularities, and potential misalignment, all of which may influence measurement readings. As in Protocol I, four measurements were obtained and averaged to determine the final specimen thickness. Given that caliper-based measurements inherently involve manual control of both contact positioning and applied pressure, the two protocols were specifically developed to capture realistic sources of user-dependent variability in thickness assessment. In Protocol III, sample thickness was measured with a Mitutoyo 547-500S digital thickness gauge, and the final value was obtained by averaging three consecutive measurements (Figure 1). To minimize experimental variability, all specimens were maintained in phosphate-buffered saline (PBS) throughout the measurement and testing procedures to preserve hydration. Measurements were performed at room temperature within a standardized time window following tissue harvesting to reduce time-dependent viscoelastic changes. Specimens were consistently oriented with the luminal surface facing upward, and care was taken to avoid pre-stretch or deformation prior to thickness measurement. The order of measurement protocols was standardized across all specimens (Protocol I, Protocol II, followed by Protocol III) to ensure consistency. To ensure consistent measurement orientation and to prevent repeated assessment of the same side or corner in Protocols I and II, one corner of each specimen was marked prior to measurement. This reference point defined the starting location and guided the sequential positioning of measurements across the specimen. Users performed measurements independently and were blinded to each other’s results. To minimize recall bias, measurements were recorded immediately and not revisited during subsequent protocols.

### 2.3 Biomechanical testing

Biomechanical testing was conducted using a BioTester® 5000 (CellScale, Waterloo, ON, Canada), fitted with two opposing BioRakes mounted on actuators to apply uniaxial tensile loading to the arterial wall (Figure 1). For each specimen, an initial gauge length of 10 mm was established, and the resulting force–displacement response was recorded. Samples were subjected to cyclic loading consisting of a 25% stretch followed by a 50% stretch, applied at rates of 1% s^-1^ and 2% s^-1^, respectively, for a total of 10 cycles each. Only data from the final cycle were used for statistical analysis to ensure mechanical stabilization of the tissue. Each loading cycle comprised 25 s of stretching followed by a 25-s recovery period. Based on the recorded data and using LabJoy 2.0 software (CellScale), the Cauchy stress (kPa) and Young’s modulus (kPa) were calculated. Cauchy stress (σ) was calculated as the ratio of the measured force to the specimen cross-sectional area (σ = F/A), where F represents the force recorded at 25% and 50% stretch, and A denotes the cross-sectional area, defined as the product of specimen width and wall thickness. Young’s modulus (E) was determined from the slope of the linear region of the stress–strain response (E = Δσ/Δε), evaluated over the strain interval corresponding to 25%–50% stretch during the final loading cycle.

### 2.4 Statistical analysis

Statistical analyses were performed using SPSS for Mac OS (version 29.0.2.0; SPSS Inc., Chicago, IL, USA). Continuous variables, including thickness, Cauchy stress, and Young’s modulus, are presented as mean ± standard deviation (SD). Differences between continuous variables were assessed using a one-way ANOVA test, followed by Tukey’s multiple comparisons test to adjust for pairwise comparisons. Comparisons were performed separately within each vessel type, with thickness measurements obtained from different protocols or users treated as independent groups for method comparison. For the purpose of method comparison, measurements obtained from different protocols or users were treated as independent groups, despite being derived from repeated assessments of the same specimens. This approach was adopted to enable direct group-wise comparisons within a unified analytical framework. However, it does not account for within-specimen correlations inherent to the study design.

## 3 Results

**FIGURE 2:**
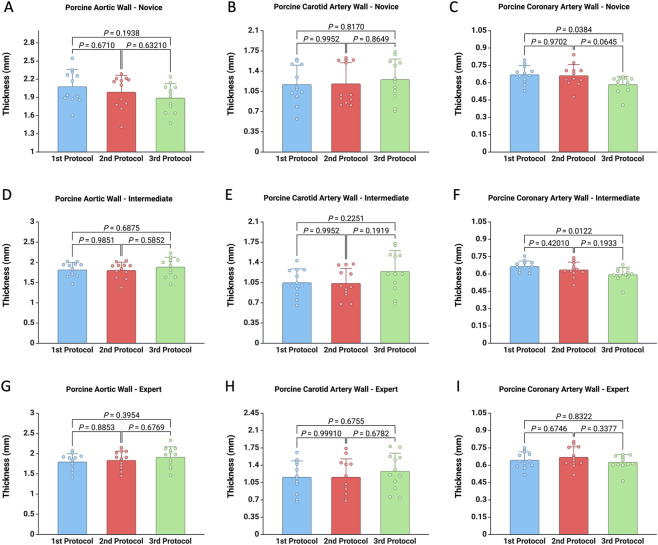
Comparison of measurements from three experimental protocols across three porcine vascular tissues (aorta, carotid, and coronary artery walls), obtained by users with novice **(A–C)**, intermediate **(D–F)**, and expert **(G–I)** experience levels.

Arterial wall thickness measurements were compared across three measurement protocols for users with novice, intermediate, and expert levels of experience, and across three vessel types: porcine aorta, porcine carotid artery, and porcine coronary artery (Figure 2; Table 1). Across vessel types, both the magnitude and statistical significance of inter-protocol differences were strongly influenced by tissue thickness and operator experience. For the porcine aorta, inter-protocol variability was minimal, with absolute differences ≤0.18 mm and relative deviations generally below 10%, and no statistically significant differences were detected at any experience level (Figures 2A,D,G). Although novice users tended to overestimate thickness with caliper-based protocols relative to the digital thickness gauge (Protocol III), intermediate and expert users showed smaller, more consistent deviations, indicating improved measurement precision with experience. Similarly, carotid artery measurements did not differ significantly across protocols for any user group (Figures 2B,E,H), despite a greater spread of values than in the aorta. Notably, intermediate users systematically underestimated caliper-based methods, with deviations of approximately 16% relative to Protocol III, suggesting that moderately thick, compliant tissues are more sensitive to methodological differences and operator-dependent factors such as contact force and positioning. In contrast, the porcine coronary artery—the thinnest vessel examined—demonstrated clear protocol-dependent effects. Significant differences were observed between Protocol I and Protocol III for both novice (p = 0.0384; Figure 2C) and intermediate users (p = 0.0122; Figure 2F), while no significant differences were detected for expert users (Figure 2I). Caliper-based protocols consistently overestimated thickness in novice and intermediate groups, with relative differences of approximately 12%–14% compared with Protocol III. These findings highlight the increased susceptibility of thin-walled tissues to compression artifacts and alignment errors inherent to manual caliper measurements.

**TABLE 1:** Quantitative comparison of arterial wall thickness measurements across protocols and user experience levels, including absolute and relative differences between caliper-based methods and digital thickness gauge measurements.

Vessel type	User experience	Protocol I (mean ± SD)	Protocol II (mean ± SD)	Protocol III (mean ± SD)	Absolut difference			%Δ		
					P1-P2	P1-P3	P2-P3	P1 vs. P2	P1 vs. P3	P2 vs. P3
Porcine aorta	Novice	2.08 ± 0.27	1.99 ± 0.27	1.89 ± 0.23	0.09	0.18	0.10	4.57%	9.95%	5.15%
	Intermediate	1.82 ± 0.17	1.81 ± 0.19	1.89 ± 0.23	0.01	−0.07	−0.08	0.76%	−3.65%	−4.38%
	Expert	1.80 ± 0.19	1.84 ± 0.22	1.92 ± 0.25	−0.04	−0.12	−0.08	−2.33%	−6.25%	−4.02%
Porcine carotid artery	Novice	1.17 ± 0.33	1.18 ± 0.36	1.26 ± 0.35	−0.01	−0.08	−0.07	−1.12%	−6.79%	−5.74%
	Intermediate	1.05 ± 0.23	1.04 ± 0.25	1.25 ± 0.35	0.01	−0.19	−0.21	1.03%	−15.73%	−16.58%
	Expert	1.16 ± 0.32	1.16 ± 0.36	1.28 ± 0.36	−0.001	−0.12	−0.12	−0.04%	−9.41%	−9.37%
Porcine coronary artery	Novice	0.67 ± 0.07	0.66 ± 0.09	0.59 ± 0.07	0.03	0.07	0.04	4.64%	11.89%	6.93%
	Intermediate	0.67 ± 0.04	0.64 ± 0.06	0.59 ± 0.06	0.001	0.08	0.07	1.16%	14.42%	13.10%
	Expert	0.64 ± 0.07	0.67 ± 0.08	0.63 ± 0.06	−0.02	0.02	0.04	−3.85%	2.80%	6.91%

Overall, measurement variability decreased with increasing operator experience across all vessel types, with the most pronounced improvement observed in the coronary artery. Variability was inversely related to vessel wall thickness and amplified among less experienced users. Across all conditions, the digital thickness gauge (Protocol III) yielded systematically lower and more consistent measurements, supporting its reduced operator dependence and superior reproducibility.

To evaluate the reproducibility of each protocol regarding the user experience, thickness measurements obtained using each protocol were compared separately for each tissue type. As shown in Supplementary Figure S1A, when applying the first protocol to the porcine aortic wall, the novice user obtained significantly greater thickness measurements compared with both the intermediate (p = 0.0181) and expert users (p = 0.0097). In contrast, no significant differences among users were observed when using the second and third protocols (Supplementary Figures S1B, C1). Likewise, for the porcine carotid artery specimen, user experience did not significantly affect measurements for any of the three protocols (Supplementary Figure S1).

**FIGURE 3:**
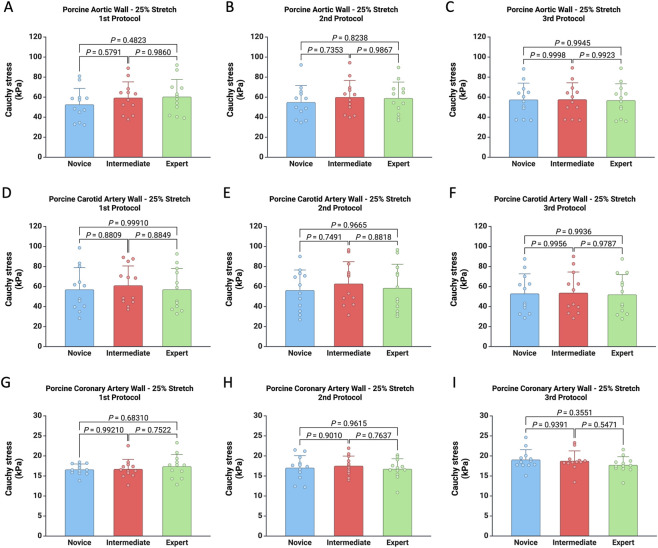
Comparison of Cauchy stress (kPa) calculated using thickness measurements from the three protocols for each user for the porcine aortic wall **(A–C)**, carotid artery wall **(D–F)**, and coronary artery wall **(G–I)** subjected to 25% stretch.

Subsequently, the specimens were subjected to uniaxial tensile loading at strains of 25% and 50% to assess their biomechanical properties. The thickness measurements obtained by each user using each protocol were then used to calculate Cauchy stress (kPa) and Young’s modulus (kPa). The mechanical response of porcine vascular tissues at 25% stretch was not influenced by operator experience, as no statistically significant differences in Cauchy stress were observed among the Novice, Intermediate, and Expert users across any of the three experimental protocols (for all p > 0.05) (Figure 3). While stress levels varied by anatomical location—with the aortic wall being the most resistant, followed by the carotid and coronary walls—the variability was consistent across the three users.

**FIGURE 4:**
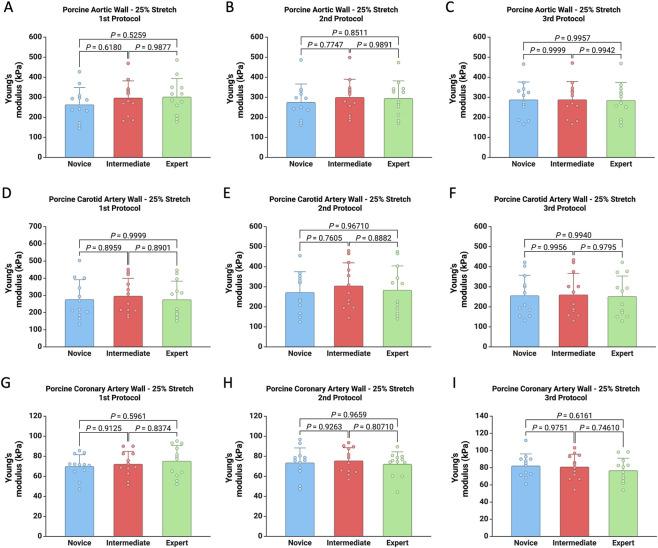
Comparison of Young’s modulus (kPa) calculated using thickness measurements from the three protocols for each user for the porcine aortic wall **(A–C)**, carotid artery wall **(D–F)**, and coronary artery wall **(G–I)** subjected to 25% stretch.

The Young’s modulus of porcine aortic, carotid, and coronary artery walls at 25% stretch remained consistent across all user experience levels, with no significant differences found between the novice, intermediate, and expert groups (Figure 4). This uniformity was maintained across three consecutive experimental protocols, suggesting that all the protocols proposed and analyzed in the current study were independent of user experience. Moreover, similar results were observed when porcine vascular tissue was subjected to 50% stretch (Supplementary Figures S2, S3).

## 4 Discussion

The main findings of this study demonstrate that the consistency of arterial wall thickness measurements improves with increasing operator experience, particularly in small vessels with thin walls, such as the coronary arteries. Novice and intermediate users exhibited protocol-dependent variability in their measurements, whereas these differences were no longer observed at the expert user. Despite this variability in raw thickness data, all three measurement protocols—whether using a digital vernier caliper or a dedicated digital thickness gauge—produced comparable biomechanical outcomes for Cauchy stress and Young’s modulus across all levels of user experience. This indicates that although operator experience and measurement technique can affect thickness measurements, the resulting biomechanical characterization of porcine aortic, carotid, and coronary arteries remains largely robust to these variations, mainly because of the high data distribution. Overall, the results support the reliability of biomechanical analyses when standardized thickness measurement protocols are used, while underscoring the importance of operator training to reduce measurement variability, particularly in thinner vascular tissues.

In our results, the clearest protocol-dependent differences were observed in the coronary artery for novice and intermediate users, whereas a user-specific difference was observed in the aorta wall under Protocol I, with the novice user measuring higher values than the intermediate and expert users. This pattern likely reflects the combined effects of tissue thickness, local flatness, and sensitivity to compression/alignment errors. Expert users likely reduce variability by applying more consistent contact force, maintaining better jaw/probe alignment, selecting more reproducible contact sites, and avoiding oblique placement or over-compression. Untrained users are more likely to compress the specimen, misalign the device, or choose slightly different local regions, which becomes particularly important in thin, compliant vessels. These interpretations are directly supported by the observed reduction in variability with increasing experience and by the gauge’s lower operator dependence.

Our findings demonstrate that while both instruments are capable of producing measurements within an acceptable range for gross anatomical assessment, depending on the protocol applied, the digital thickness gauge exhibited superior repeatability and reduced operator-dependent variability, especially when tissue thickness was small. These differences have important practical implications for laboratories conducting *ex vivo* vascular characterization, where consistency and reproducibility are essential for minimizing methodological noise and improving statistical power in mechanical testing datasets (Cardiovascular Solid Mechanics, 2025; Holzapfel and Ogden, 2003; Fung, 1993).

The superior performance of the digital thickness gauge aligns with previous studies emphasizing the importance of consistent contact pressure and stable probe alignment when measuring compliant biological tissues (Silver et al., 2001; Carew et al., 1968; Holzapfel et al., 2012). The gauge’s spring-loaded mechanism appears to facilitate more uniform force application, thereby mitigating tissue compression artifacts—a known source of systematic error when using calipers on soft tissues (Nielsen et al., 1991; Vorp et al., 2003). In contrast, the Vernier caliper, designed primarily for rigid materials, requires the operator to actively control the applied force, making it more susceptible to inter- and intra-user variability, particularly with thin arterial specimens (Corporation, 2019).

Another key observation was that the digital thickness gauge reduced measurement time, thereby improving the overall efficiency of biomechanical analysis workflows. Measurement efficiency is especially relevant in *ex vivo* vascular testing, where numerous samples must be processed rapidly to prevent tissue dehydration, viscoelastic relaxation, enzymatic degradation, or temperature-related changes in mechanical response (O’Rourke et al., 2002; Sommer et al., 2010). Faster, standardized measurement protocols may therefore indirectly improve data quality by limiting exposure to time-dependent confounding factors.

An additional consideration is the influence of tissue handling technique. While the thickness gauge reduced operator dependence, residual variability may still arise from differences in tissue hydration, sample orientation, axial stretch, and preconditioning history—factors that are well documented to affect vascular mechanical behavior and apparent wall thickness (Sacks and Sun, 2003).

These findings are consistent with recent studies on vascular tissue measurement, particularly those using porcine venous and arterial specimens. In that work, the digital thickness gauge (Mitutoyo 547-500S) demonstrated superior consistency, with repeated measurements showing no significant differences across three consecutive trials (p = 0.953, p = 0.742, and p = 0.897) and minimal variability regardless of examiner experience. In contrast, Vernier caliper protocols—especially those involving multi-point or bilateral measurements—produced statistically significant differences between sequential measurements (often p < 0.05) and systematically underestimated initial thickness values relative to subsequent readings [30]. Such discrepancies can introduce errors of approximately 10%–15% in arterial wall thickness estimation, potentially leading to misleading interpretations of arterial stiffness, stress distribution, and failure properties (Vorp et al., 2003; Raghavan et al., 1996).

From a user-experience perspective, the results strongly favored the digital thickness gauge. Its ergonomic design, fixed contact-pressure control, and one-handed operation reduced reliance on operator skill and training, thereby minimizing measurement variability and improving reproducibility across laboratories. By contrast, Vernier calipers require careful alignment, bilateral contact control, and visual verification, increasing the likelihood of misalignment and misinterpretation—particularly when measuring thin, deformable vascular tissues (Corporation, 2019).

Accurate wall thickness measurement is critical for computing biomechanical parameters such as Cauchy stress, circumferential strain, and Young’s modulus derived from tensile or inflation-extension testing. Even modest inaccuracies in thickness measurement can significantly distort stress–strain curves and bias biomechanical interpretation (Humphrey and Holzapfel, 2012; Sokolis, 2007; S and hadwick, 1999). Employing a thickness gauge, therefore, improves internal validity and reduces error propagation in biomechanical models that depend on precise geometric inputs.

Our results mirror prior findings in soft-tissue metrology, where compliant tissues are more reliably measured using thickness gauges or controlled-force micrometers, whereas heterogeneous or porous tissues (e.g., aneurysmal intraluminal thrombus) may require alternative approaches, including imaging-based or sectioning methods (Vorp et al., 2003; Di Martino et al., 2006). Moreover, extensive literature on arterial intima–media thickness assessment via ultrasound highlights the importance of reproducible, standardized measurement protocols and reduced operator bias, further reinforcing the value of consistency over manual estimation (Stein et al., 2008; Touboul et al., 2012; Bots et al., 1997).

However, both instruments demonstrate inherent limitations. Neither device provides real-time, *in situ* wall thickness measurements comparable to those obtained with high-resolution imaging modalities such as ultrasound, optical coherence tomography (OCT), or micro–computed tomography (micro-CT). Although these techniques offer superior spatial resolution and eliminate direct tissue manipulation, they remain costly, technically demanding, and less accessible for many experimental biomechanics laboratories (Chiu et al., 2008; Rezakhaniha et al., 2012; Łagan and Liber-Kneć, 2020). Within this context, the digital thickness gauge represents a practical compromise between affordability, ease of use, and measurement reliability.

This study utilized porcine arterial specimens at room temperature under *ex vivo* conditions. Extrapolation to *in vivo* human arteries must therefore consider physiological factors such as smooth muscle tone, pulsatile loading, blood pressure–dependent deformation, and temperature effects on tissue compliance (Vlachopoulos et al., 2011; Humphrey and Delange, 2004; Laurent et al., 2006). Future studies correlating caliper- and gauge-based measurements with imaging modalities such as high-resolution ultrasound or OCT would further validate measurement accuracy and clinical relevance. Additionally, systematic evaluation of contact-force calibration, tissue stiffening, and ambient humidity effects would help refine standardized protocols for vascular thickness measurement.

Although protocol-dependent differences in thickness measurements were observed, particularly in thinner vessels and among less experienced users, these variations did not translate into statistically significant differences in derived biomechanical parameters. This is likely because the magnitude of thickness variation was relatively small compared with the inherent variability in force–displacement response between specimens. As a result, the influence of geometric measurement differences on calculated stress and stiffness was limited under the present experimental conditions.

To place the present findings in context, it is essential to acknowledge the substantial methodological variability across studies in both biomechanical testing and arterial wall thickness measurement (Dwivedi et al., 2023; Pukaluk et al., 2022; de Beaufort et al., 2018; Bechsgaard et al., 2018; Arbănaşi et al., 2024; Peña et al., 2022; Mureşan et al., 2024). Prior investigations of porcine arterial tissues, including those conducted by Sachin Kumar’s group (Dwivedi et al., 2023), have frequently utilized multiaxial bulge or inflation testing to better approximate physiologically relevant loading conditions. Such approaches enable robust characterization of biaxial stress–strain behavior as well as spatial heterogeneity within the vessel wall (Dwivedi et al., 2023). In contrast, a large proportion of the literature relies on uniaxial tensile testing of arterial strips with constitutive models applied to capture the nonlinear mechanical response (Arbănaşi et al., 2024). Although widely adopted due to experimental simplicity, uniaxial testing does not fully represent the coupled, anisotropic behavior of vascular tissues and may introduce directional bias in stiffness estimates (Pukaluk et al., 2022; de Beaufort et al., 2018; Bechsgaard et al., 2018). Equally important, yet often underappreciated, is the lack of standardization in thickness measurement protocols, which represents a significant source of variability. For instance, some studies employ digital micrometers with controlled contact forces to limit tissue compression and enhance measurement reproducibility (Ion et al., 2024a; Ion et al., 2024b), whereas others rely on manual measurements obtained during specimen preparation with a digital caliper (Arbănaşi et al., 2024; Mureşan et al., 2024). Taken together, these methodological disparities suggest that reported differences in stress and stiffness across studies may not solely reflect biological variability but are also influenced by experimental design—particularly the choice of loading configuration and, critically, the approach used to quantify wall thickness (Famaey et al., 2026). An additional potential source of bias arises from variability in specimen storage conditions prior to mechanical testing (Arbănaşi and Chirilă, 2026; Alloisio et al., 2024; Lin et al., 2022; Eliathamby et al., 2022; Peterson et al., 2025). In most experimental protocols, tissues are stored for short-to mid-term periods under controlled conditions before analysis, which may inadvertently influence their structural and mechanical properties (Arbănaşi and Chirilă, 2026; Alloisio et al., 2024). Conversely, some groups advocate the use of freshly harvested specimens—typically tested within 4 h (Eliathamby et al., 2022) to 24 h (Peterson et al., 2025) — to minimize storage-induced alterations and better preserve native biomechanical behavior. This lack of consensus in handling protocols further underscores the challenges in comparing results across studies and highlights the need for standardized preservation and testing procedures.

As a practical guideline, compliant soft tissues with millimeter-scale thickness should preferentially be measured using a digital thickness gauge with controlled contact force, particularly when specimens are thin, highly deformable, or evaluated by multiple operators. When a digital vernier caliper is employed, measurement variability should be minimized through strict standardization, including use of a single trained operator, adherence to a predefined multi-point measurement protocol, maintenance of perpendicular jaw alignment, application of minimal compressive force, and preservation of continuous specimen hydration.

The present study has several limitations that warrant consideration. First, our experimental investigation included twelve specimens for each type of porcine arterial tissue. Although the observed measurement biases in wall thickness did not ultimately influence the derived biomechanical properties, the relatively small sample size and the considerable data variability should be considered when interpreting the results. Based on our findings, we recommend that users employ a digital thickness gauge to enhance measurement reproducibility and consistency. However, if a digital vernier caliper is used, measurements should be performed by a highly experienced operator. Secondly, our analysis was restricted to healthy and uniform porcine vascular tissue. Future studies should evaluate both measurement devices using normal and pathological human arterial tissues to corroborate and extend the conclusions of the current work. Third, the study lacked an independent gold-standard reference for arterial wall thickness measurement. Although reproducibility and inter-protocol agreement were assessed, absolute measurement accuracy could not be definitively established. Other limitations include potential tissue-compression artifacts, which are inherent to both instruments tested. Thus, the results of the current study should not be directly extrapolated to small-animal vessels, such as mouse or rat aorta, whose wall thickness is in the micrometer range. In such settings, contact-based caliper or gauge measurements may be limited by probe size, device resolution, and tissue compression. While the digital thickness gauge employs a spring-loaded mechanism designed to standardize contact force, some degree of tissue deformation is unavoidable when measuring compliant biological materials. This effect may be particularly pronounced in thinner vessels such as coronary arteries, potentially leading to systematic underestimation of wall thickness and influencing derived biomechanical parameters. A principal limitation of this study concerns the statistical treatment of repeated measurements. As identical specimens were evaluated across multiple protocols and operators, the resulting observations are inherently non-independent. However, for analytical simplicity and to maintain comparability across protocols and users within a unified framework, the data were modeled as independent groups rather than employing a repeated-measures or mixed-effects approach. While this strategy facilitated straightforward group-wise comparisons, it does not account for within-specimen correlations and may have led to an underestimation of variability, thereby influencing the risk of both type I and type II errors. Accordingly, the absence of statistically significant differences in the assessed biomechanical parameters should not be interpreted as evidence of equivalence between measurement methods, but rather as a lack of detectable differences within the constraints of the applied analytical framework. Future studies using larger sample sizes and mixed-effects modeling approaches may further refine these findings.

## 5 Conclusion

In conclusion, this study demonstrates that the method used to measure arterial wall thickness influences measurement reproducibility, particularly for thin-walled vessels such as coronary arteries and when measurements are performed by less experienced users. Operator experience significantly reduced variability when using a digital vernier caliper, whereas the dedicated digital thickness gauge provided more consistent and user-independent measurements across all vessel types and experience levels. Nevertheless, to minimize methodological noise, reduce operator bias, and improve reproducibility—especially in small or highly compliant vessels—we recommend a digital thickness gauge as the preferred tool for arterial wall thickness determination in *ex vivo* biomechanical studies. These findings should be interpreted in the context of the study design and its statistical limitations, particularly regarding the handling of repeated measurements.

## Data Availability

The raw data supporting the conclusions of this article will be made available by the authors, without undue reservation.
