# Supplementary material for: Analysis of digital vernier caliper versus digital thickness gauge consistency and user experience in measuring arterial wall thickness: implications for biomechanical assessment
Source: Front Bioeng Biotechnol. 2026 May 12;14:1791599. doi: 10.3389/fbioe.2026.1791599 (PMC13201447; doi:10.3389/fbioe.2026.1791599)
Supplement: Supplementary file 1 [file Supplementaryfile1.docx]

Supplementary Material

# Supplementary Figures and Tables

## Supplementary Figures


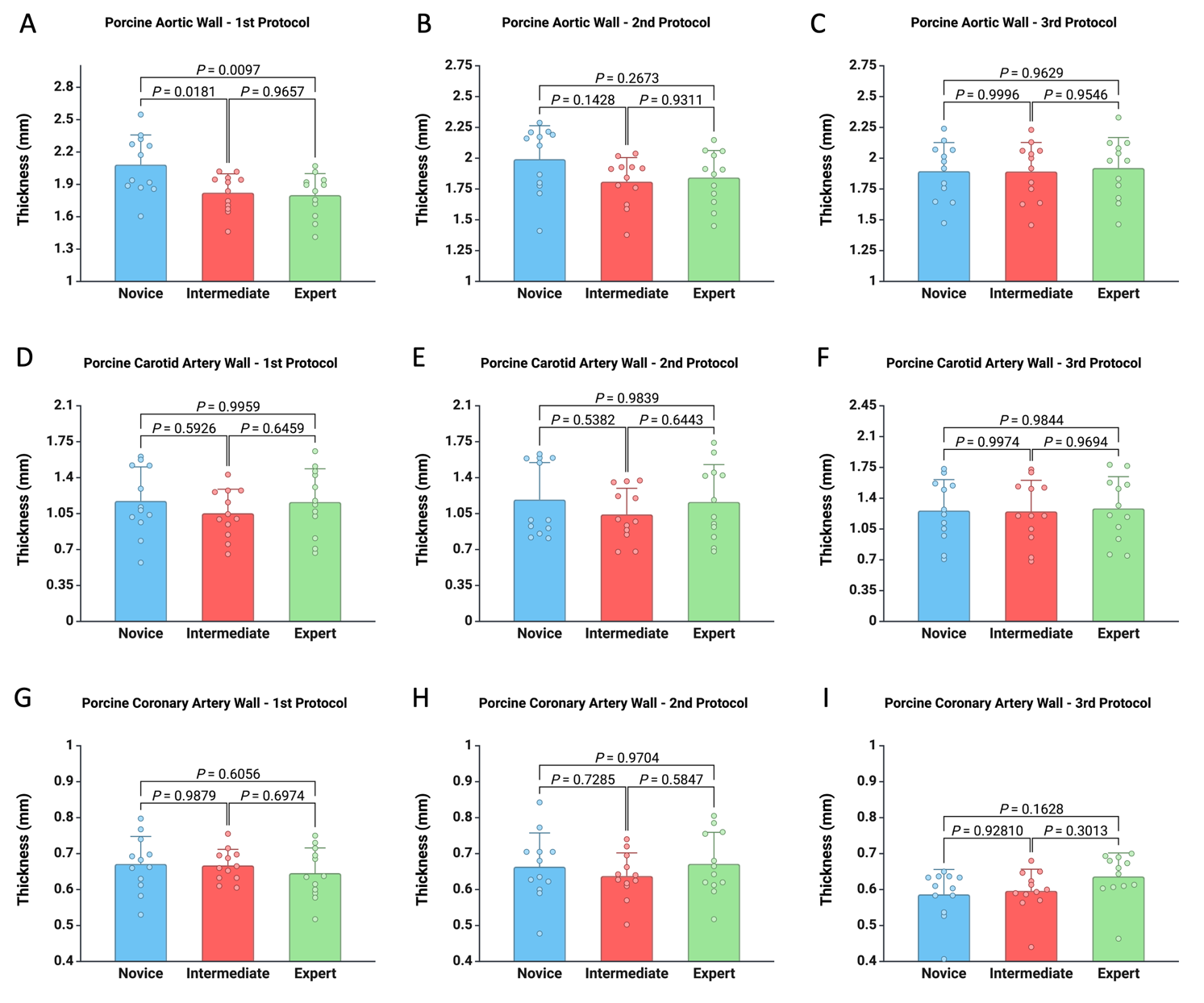


**Supplementary Figure 1.** Comparison of measurements obtained by three users across all the protocols, on the porcine aortic wall (A)–(C), porcine carotid artery wall (D)–(F), and porcine coronary artery wall (G)–(I).

**
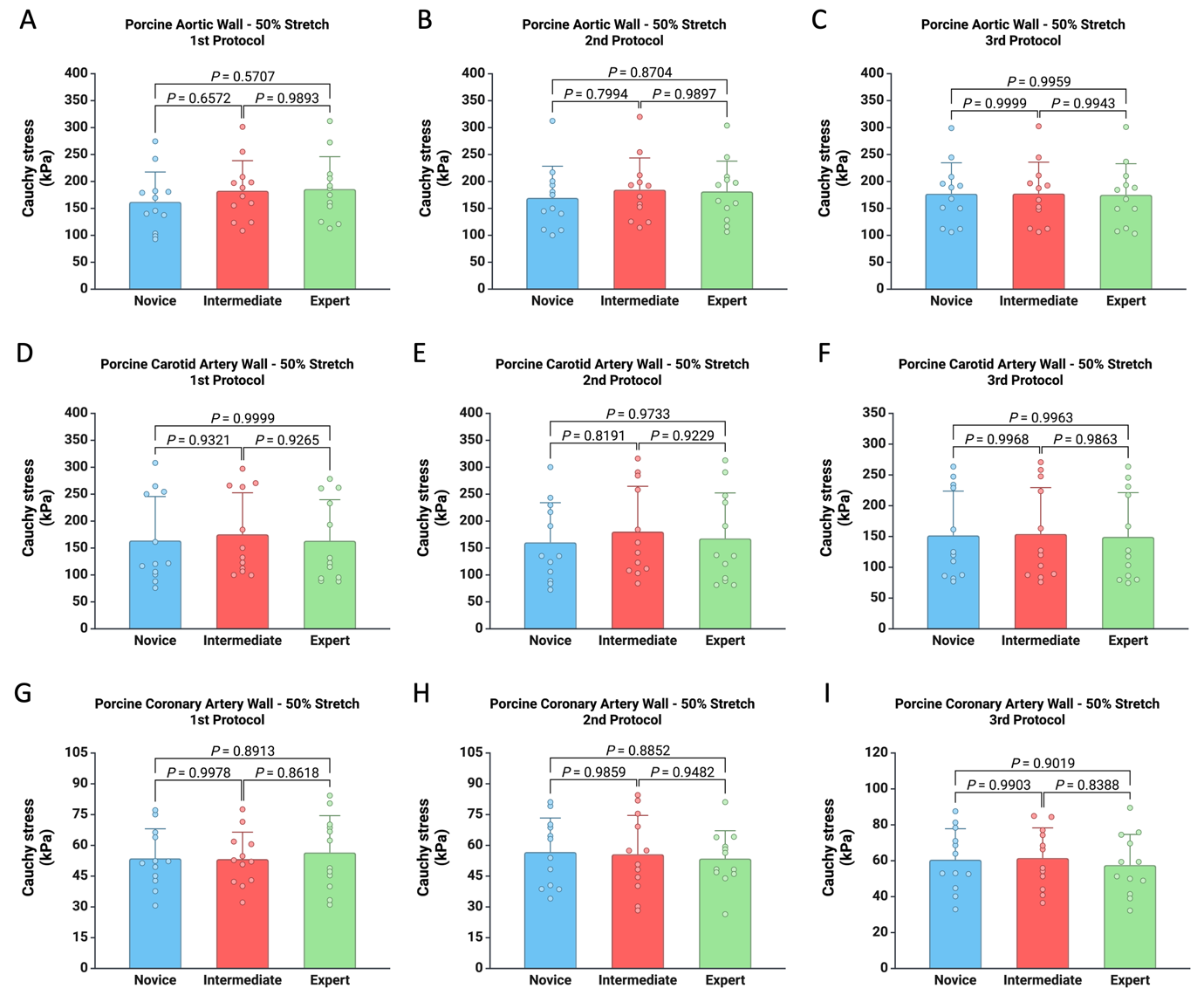
**

**Supplementary Figure 2.** Comparison of Cauchy stress (kPa) calculated using thickness measurements from the three protocols for each user for the porcine aortic wall (A)–(C), carotid artery wall (D)–(F), and coronary artery wall (G)–(I) subjected to 50% stretch.

**
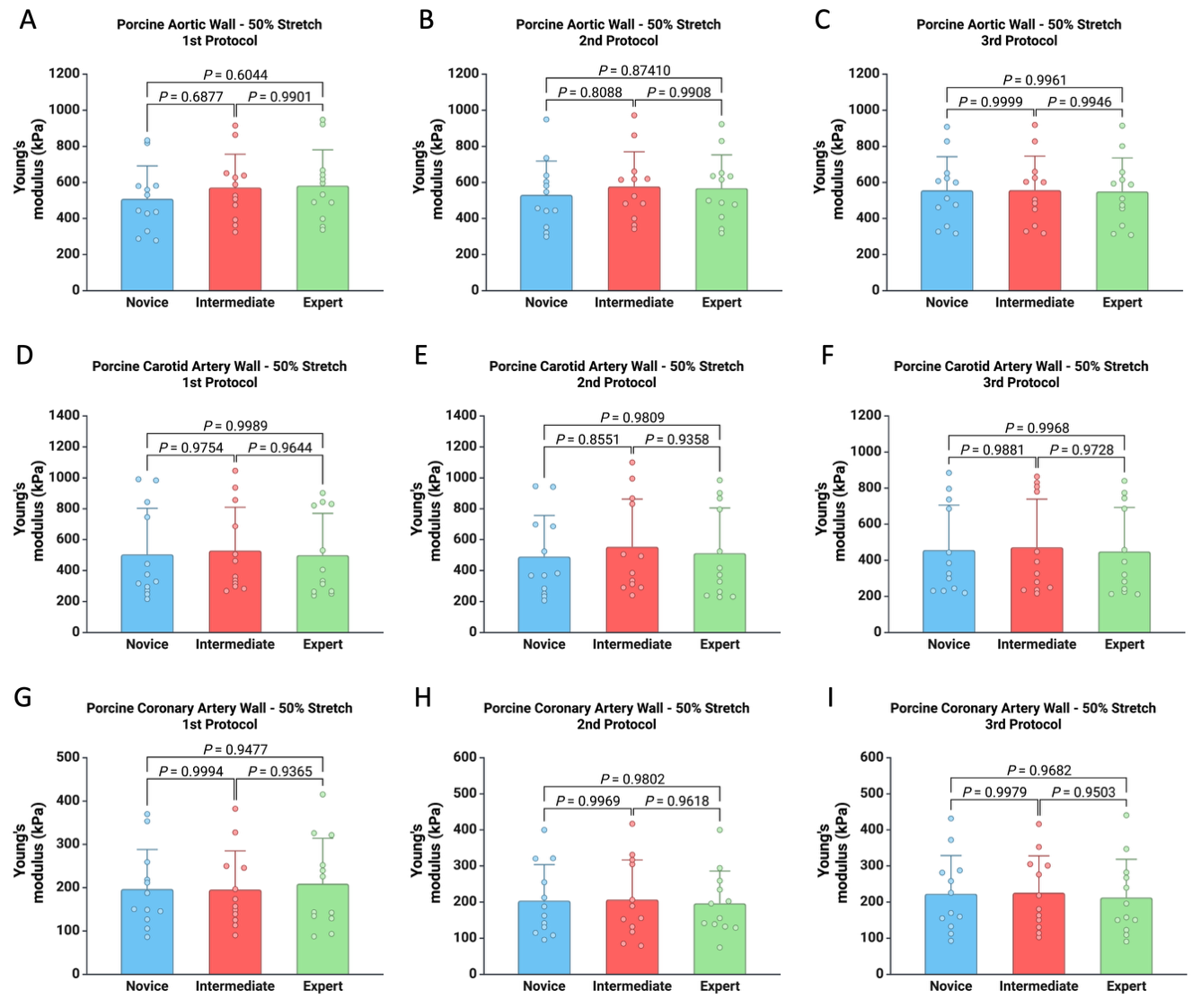
**

**Supplementary Figure 3.** Comparison of Young’s modulus (kPa) calculated using thickness measurements from the three protocols for each user for the porcine aortic wall (A)–(C), carotid artery wall (D)–(F), and coronary artery wall (G)–(I) subjected to 50% stretch.
